# Comparison of 3-level anterior cervical discectomy and fusion and open-door laminoplasty in cervical sagittal balance: A retrospective study

**DOI:** 10.3389/fsurg.2022.937479

**Published:** 2022-09-12

**Authors:** Wenhao Wang, Yixue Huang, Zhikai Wu, Xiayu Hu, Pan Xiang, Hao Liu, Huilin Yang

**Affiliations:** ^1^ Department of Orthopaedic Surgery, The First Affiliated Hospital of Soochow University, Suzhou, China; ^2^ Orthopaedic Institute, Medical College, Soochow University, Suzhou, China

**Keywords:** 3-level anterior cervical discectomy, open-door laminoplasty, cervical sagittal balance, cervical lordosis, clinical efficacy

## Abstract

**Objective:**

To compare the clinical efficacy and radiological outcomes of 3-level anterior cervical discectomy and fusion (ACDF) and open-door laminoplasty (LP).

**Methods:**

A total of 74 patients from January 2017 to January 2020 were enrolled in this retrospective study. There were two groups. Group A (30 cases) received 3-level ACDF, while Group B (44 cases) received open-door LP. Clinical evaluation included perioperative parameters, Neck Disability Index (NDI), and Japanese Orthopaedic Association (JOA) scores. Radiological evaluation included cervical curve depth (CCD), C2–7 angle, C2–7 sagittal vertical axis (cSVA), C7 slope (C7S), and T1 slope (T1S).

**Results:**

Perioperative parameters such as blood loss, drainage volume after surgery, and hospital stay of patients in Group A were significantly less than those in Group B (*P* < .001). NDI scores decreased and JOA scores increased significantly after surgery in both groups (*P* < .05). There was a significant difference in both scores postoperatively and at 1 month after surgery between the two groups (*P* < .05). CCD and C2–7 angle of Group A increased significantly postoperatively at 1 month after surgery and at final follow-up (FFU) (*P* < .05). There was a significant difference in CCD and the C2–7 angle between the two groups postoperatively at 1 month after surgery and at FFU (*P* < .05). T1S increased significantly in Group A postoperatively and at 1 month after surgery (*P* < .05).

**Conclusion:**

3-level ACDF and open-door LP achieved favorable clinical outcomes and ACDF benefited patients in the early stage of rehabilitation. Compared with open-door LP, 3-level ACDF had advantages of reconstructing cervical lordosis with increased CCD and C2–7 angle.

## Introduction

Cervical spine surgery can be generally divided into an anterior approach and a posterior approach. Anterior cervical discectomy and fusion (ACDF), one of the typical anterior surgical methods, was first put forward by Smith and Robinson and has a history of over half a century (1). It aims to decompress the spinal cord and nerves in order to relieve the symptoms of diseases such as cervical spondylosis and spine cord injury. With the help of plate fixation, ACDF has advantages of restoring intervertebral height and maintaining cervical spine stability (2, 3). However, it also has complications such as loosening of internal fixation, the injury of the esophagus and laryngeal nerve, and axial or root symptoms (4, 5). In addition, although the safety and clinical efficacy of 3-level ACDF have been demonstrated, more complications have been reported in multi-level ACDF (6). Cervical laminoplasty (LP) is one of the typical posterior surgical methods and has been developed as an alternative to cervical laminectomy. It has the merit of widening the spinal canal and relieving compression of the spinal cord caused by cervical spondylosis or spine cord injury (7). There are two main techniques: Hirabayashi’s open-door LP and Kurokawa’s double-door LP. Studies have found that there was no significant difference in clinical outcomes between open-door and double-door LP. LP has complications such as the injury of the spinal cord and vertebral artery, spinal deformity, and C5 palsy (7).

In recent years, the relationship between cervical spine surgery and cervical sagittal balance (CSB) has become a hot spot. CSB shows how the cervical spine is postured in the sagittal section and can be quantified by measuring relevant parameters such as the C2–7 angle, sagittal vertical alignment (SVA), C7, and T1 slope (8). Cervical spine surgery can be further assessed by analyzing CSB and comparing the perioperative data of patients. However, there are few articles on cervical spine surgery from a CSB perspective. Studies on CSB may help surgeons to view cervical spine surgery from a biomechanical perspective and improve their understanding of the impact of CSB on surgery and diseases (9). In addition, some studies have compared the anterior approach and posterior approach of cervical spine surgery and reported their safety and clinical efficacy (7, 10). However, studies on the comparison of 3-level ACDF and open-door LP are relatively rare. Therefore, this retrospective comparative study was conducted to compare the effect of 3-level ACDF and open-door LP on clinical evaluation and radiological outcomes from the perspective of CSB.

## Methods

### Patients

A total of 74 patients who were admitted to the orthopedics department of our hospital from January 2017 to January 2020 were enrolled in this retrospective study. The inclusion criteria were (1) symptoms and signs of cervical spondylotic myelopathy or spinal cord injury, (2) symptoms and signs not responsive to conservative treatments, (3) age over 18, (4) nerve root or spinal cord compression confirmed by magnetic resonance imaging (MRI), (5) at least three contiguous levels between C3 and C7 involved, and (6) patients treated with 3-level ACDF or open-door LP. The conservative treatments mentioned above required taking drugs such as anti-inflammatory drugs and training cervical muscle for over 3 months. “Not responsive” meant: (1) Symptoms and signs did not improve or even got worse after the conservative treatment protocol. (2) Severe spinal stenosis (>50%), cervical instability, or cervical kyphosis was observed during the treatment period. The exclusion criteria were (1) preoperative dysphagia and severe cervical malformation, (2) history of invasive malignant tumor and tuberculosis, (3) history of cervical spine surgery, (4) evidence of systemic or local infection, and (5) patients who were unable to complete at least 1-year follow-up. Informed consent was obtained from all the patients involved in this study.

### General information

The included patients were divided into two groups. Group A (30 cases) received 3-level ACDF, while Group B (44 cases) received open-door LP. Demographic data of the two groups were recorded. All the surgical procedures were performed by the same group of surgeons. Patients’ data and images were obtained from the electronic medical record management system of our hospital. None of the patients were lost in the follow-up. This study was carried out with the approval of ethics committee of our hospital.

### Clinical evaluation

The perioperative parameters of the two groups, such as operative time, blood loss, drainage volume at one day after surgery, and hospital stay, were all recorded and analyzed. For the measurement of clinical outcomes, all patients filled up the Neck Disability Index (NDI) and Japanese Orthopaedic Association (JOA) questionnaires preoperatively, postoperatively, 1 month after surgery, and at the final follow-up (FFU, at least 1 year). The NDI score ranged from 0 (no disability) to 50 (totally disabled), indicating the severity of disability and patients’ recovery after surgery. The cervical JOA score ranged from 0 (the severest dysfunction) to 17 (no dysfunction) and scored from three aspects that indicated patients’ dysfunction in motor, sensory, and bladder function.

### Radiological evaluation

Computed tomographic (CT) scan and MRI were performed before surgery and the lateral X-rays were performed preoperatively, postoperatively, 1 month after surgery, and at FFU. Cervical curve depth (CCD), C2–7 angle, C2–7 sagittal vertical axis (cSVA), C7 slope (C7S), and T1 slope (T1S) were all measured and analyzed. CCD is the maximum distance between the line connecting the posterior superior edge of C2 to the posterior inferior corner of C7 and the posterior cervical curvature curve. The C2–7 angle was measured as the angle between the tangent lines of the lower endplates of the axis and C7. cSVA is the distance from the posterosuperior corner of C7 to a vertical line from the center of the C2 vertebra. C7S is the angle formed between a horizontal line and the superior endplate surface of C7. T1S is the angle between the upper endplate of T1 and the horizontal line. The lateral radiograph for measuring radiographic parameters is shown in Figure 5.

### Statistical analysis

SPSS 22.0 statistical software (SPSS Inc.) was used for data processing. The measurement data were shown as mean ± standard deviation. The *t*-test was used for a comparison of means, while the *χ*^2^ test was used for analyzing categorical variable data. There was a statistically significant difference when *P* < .05.

## Results

### Demographics

Demographic data are presented in Table 1. There were 30 patients in Group A and 44 in Group B. The average age was 59.87 ± 8.84 years in Group A and 55.91 ± 10.76 years in Group B. Male patients were more than female patients in both groups and there was no statistical difference in terms of gender composition (*P* > .05). The BMI was 23.60 ± 4.94 and 24.86 ± 3.65 kg/m^2^, respectively. A total of 20.00% of patients in Group A and 6.82% of patients in Group B underwent cervical spine surgery after trauma, and there was no significant difference between the two groups (*P* > .05). The number of patients with hypertension, diabetes, and smoking was also shown in the table. However, there was no statistically significant difference (*P* > .05). The average follow-up period was 15.93 ± 2.86 months and 16.20 ± 3.43 months, respectively, and no significant difference was observed between the two groups (*P* > .05).

**Table 1:** Demographic data and perioperative parameters of two groups.

Demographic data	Total	Group A	Group B	*P*-value
Cases (*n*)	74	30	44	
Age (years)	57.51 ± 10.09	59.87 ± 8.84	55.91 ± 10.76	0.100
Gender (male/female)	47/27	16/14	31/13	0.133
BMI (kg/m^2^)	24.35 ± 4.21	23.60 ± 4.94	24.86 ± 3.65	0.210
Trauma history (*n*)	9 (12.16%)	6 (20.00%)	3 (6.82%)	.089
Comorbidity (*n*)				
Hypertension	21 (28.38%)	6 (20.00%)	15 (34.09%)	0.187
Diabetes	8 (10.81%)	4 (13.33%)	4 (9.09%)	0.564
Smoking	14 (18.92%)	5 (16.67%)	9 (20.45%)	0.683
Follow-up (months)	16.09 ± 3.20	15.93 ± 2.86	16.20 ± 3.43	0.723
Parameters	Group A	Group B	*P*-value
Operative time (min)	206.43 ± 75.46	182.09 ± 63.54		0.138
Blood loss (ml)	109.33 ± 92.32	238.18 ± 156.88		<.001*
Drainage volume** (ml)	41.00 ± 24.58	133.18 ± 64.07		<.001*
Hospital stay (days)	12.37 ± 2.75	17.64 ± 6.36		<.001*

BMI, body mass index.

* Significance between the two groups, *P* < .05.

** Drainage volume 1 day after surgery.

### Clinical outcomes

**Figure 1:**
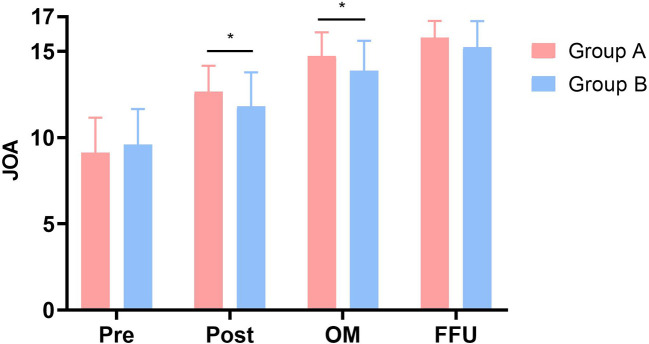
Comparison of JOA scores between two groups. Pre, preoperative; Post, postoperative; OM, one month after surgery; FFU, final follow-up. *Significance between the two groups, *P* < .05.

**Figure 2:**
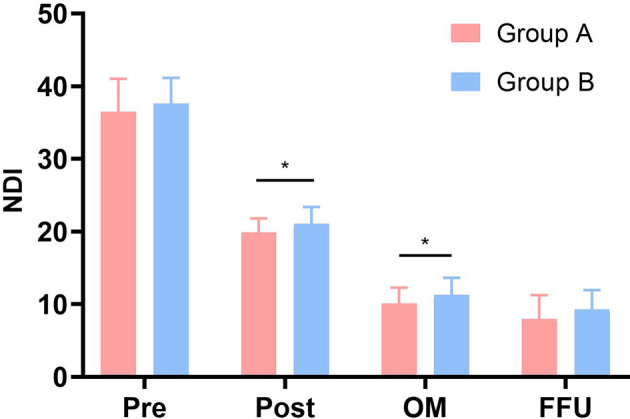
Comparison of NDI scores between two groups. Pre, preoperative; Post, postoperative; OM, one month after surgery; FFU, final follow-up. *Significance between the two groups, *P* < .05.

Perioperative parameters are also given in Table 1. The operative times were 206.43 ± 75.46 and 182.09 ± 63.54 min, respectively, which showed no significant difference (*P* > .05). However, blood loss, drainage volume at one day after surgery, and hospital stay of patients in Group A were significantly less than those in Group B (*P* < .001). JOA and NDI scores are presented in Figures 1, 2. Relevant data and the *P*-value are presented in Table 2. NDI scores significantly decreased and JOA scores significantly increased postoperatively at 1 month after surgery and at FFU compared with preoperative scores (*P* < .05). Furthermore, there was a significant difference in both scores postoperatively and at 1 month after surgery between the two groups (*P* < .05). However, no statistically significant difference was observed in NDI and JOA scores preoperatively and at FFU (*P* > .05).

**Table 2:** Comparison of NDI and JOA scores between the two groups.

	Pre	Post	OM	FFU
NDI				
Group A	36.47 ± 4.56	19.87 ± 1.93	10.10 ± 2.20	8.00 ± 3.25
Group B	37.61 ± 3.55	21.05 ± 2.33	11.27 ± 2.37	9.32 ± 2.62
*P*-value	0.229	.025*	.035*	.058
JOA				
Group A	9.13 ± 2.01	12.67 ± 1.49	14.73 ± 1.36	15.80 ± 0.96
Group B	9.59 ± 2.06	11.80 ± 1.98	13.89 ± 1.73	15.25 ± 1.50
*P*-value	0.347	.045*	.028*	.080

Pre, preoperative; Post, postoperative; OM, one month after surgery; FFU, final follow-up; NDI, Neck Disability Index; JOA, Japanese Orthopaedic Association.

* Significance between the two groups, *P* < .05.

### Radiological outcomes

**Figure 3:**
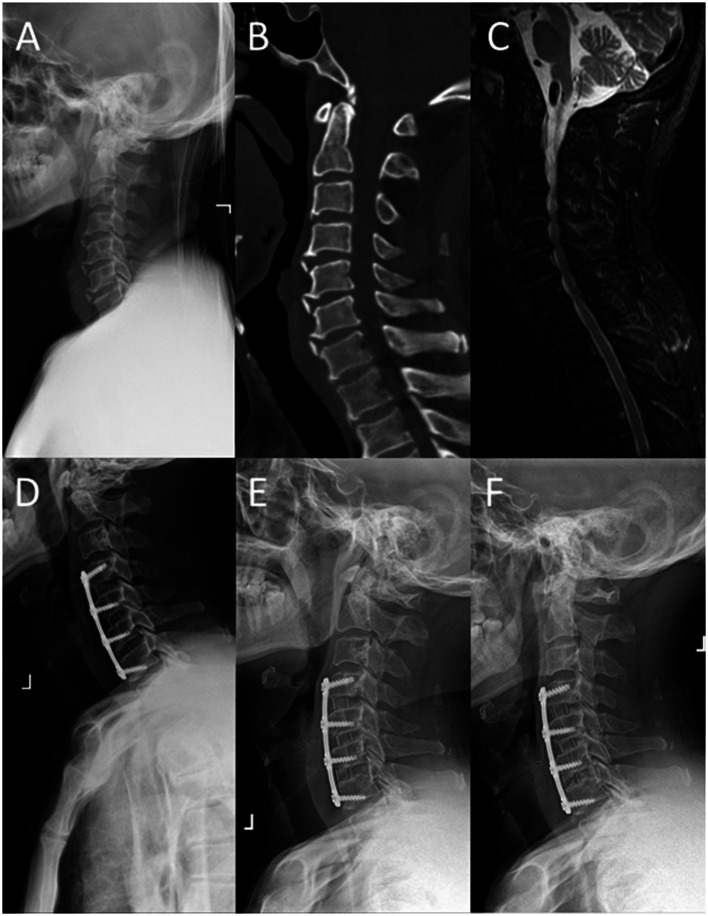
A 52-year-old male was treated with 3-level ACDF. (**A**) X-ray lateral view before surgery; (**B**) sagittal CT scan before surgery; (**C**) sagittal fat-suppressed sequence in MRI before surgery; (**D**) postoperative X-ray lateral view; (**E**) X-ray lateral view at 1 month after surgery; and (**F**) X-ray lateral view at FFU.

**Figure 4:**
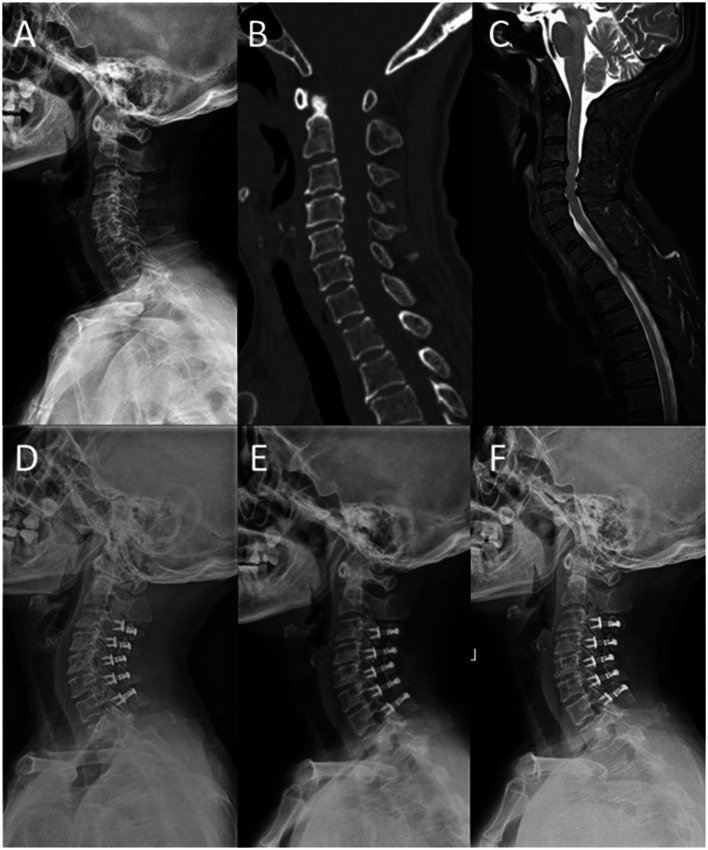
A 65-year-old female was treated with open-door laminoplasty. (**A**) X-ray lateral view before surgery; (**B**) sagittal CT scan before surgery; (**C**) sagittal fat-suppressed sequence in MRI before surgery; (**D**) postoperative X-ray lateral view; (**E**) X-ray lateral view at 1 month after surgery; and (**F**) X-ray lateral view at FFU.

The radiographic data of typical cases treated with 3-level ACDF and open-door LP are shown in Figures 3, 4, respectively. There were six parts in each figure, and these were X-ray lateral view before surgery, CT scan before surgery, fat-suppressed sequence in MRI before surgery, X-ray lateral view postoperatively, X-ray lateral view at 1 month after surgery, and X-ray lateral view at FFU. In Figures 3, 4, the compression of the cervical spinal cord was observed in MRI before surgery. After 3-level ACDF or LP, internal fixation was in place in the plane of sagittal postoperatively, 1 month after surgery, and at FFU. The CCD of both groups is shown in Figure 6. Compared with the preoperative results, the CCD of Group A increased significantly postoperatively at 1 month after surgery and at FFU (*P* < .05). However, the CCD of Group B showed no significant difference between the preoperative period and the postoperative period (*P* > .05). In addition, there was a significant difference in CCD between the two groups postoperatively at 1 month after surgery and at FFU (*P* < .05).

**Figure 5:**
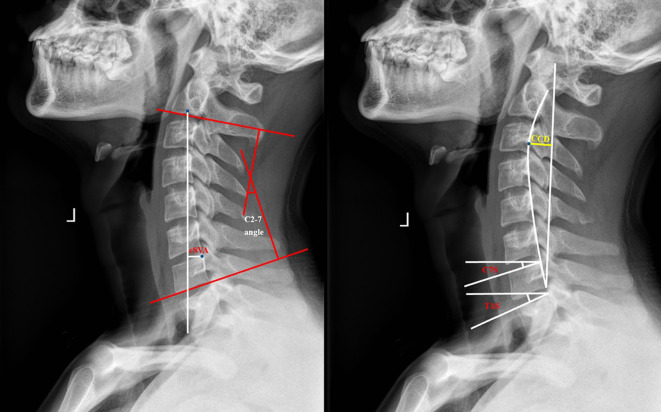
Lateral radiograph for measuring radiographic parameters. cSVA, C2-7 sagittal vertical axis; CCD, cervical curve depth; C7S, C7 Slope; T1S, T1 Slope.

**Figure 6:**
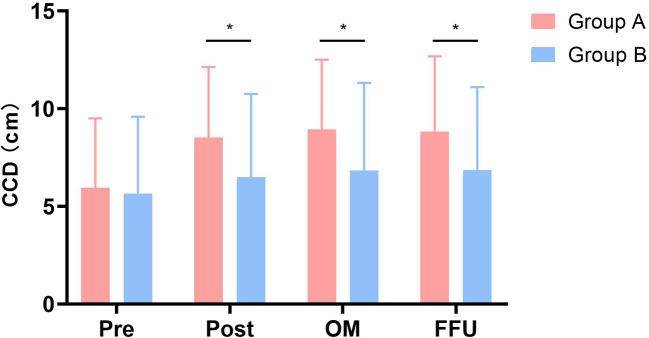
Comparison of cervical curve depth (CCD) between two groups. Pre, pre-operative; Post, postoperative; OM, one month after surgery; FFU, final follow-up. *Significance between the two groups, *P* < 0.05.

A comparison of the C2–7 angle and cSVA is given in Table 3. Compared with preoperative data, the C2–7 angle increased significantly in Group A postoperatively at 1 month after surgery and at FFU (*P* < .05). There was a significant difference in the C2–7 angle between the two groups postoperatively at 1 month after surgery and at FFU (*P* < .05, *P* < .01, *P* < .01, respectively). In terms of cSVA, no significant difference was observed in both groups. C7S and T1S are shown in Table 4. Compared with the preoperative results, T1S increased significantly in Group A postoperatively and at 1 month after surgery (*P* < .05). C7S in Group A and T1S in Group B also slightly increased after surgery. However, there was no significant difference in C7S and T1S between the two groups during the perioperative period and the follow-up.

**Table 3:** Comparison of the C2–7 angle and cSVA between the two groups.

	Pre	Post	OM	FFU
C2–7 angle (°)				
Group A	13.09 ± 7.79	18.86 ± 7.04***	20.46 ± 7.42***	20.38 ± 8.02***
Group B	14.02 ± 7.80	15.21 ± 6.47	15.37 ± 8.15	14.57 ± 8.24
*P*-value	0.617	.024*	.008**	.004**
cSVA (cm)				
Group A	2.38 ± 1.03	2.58 ± 0.78	2.35 ± 0.68	2.15 ± 0.76
Group B	2.10 ± 1.10	2.34 ± 0.89	2.00 ± 1.05	1.88 ± 0.94
*P*-value	0.270	0.230	.087	0.193

Pre, preoperative; Post, postoperative; OM, one month after surgery; FFU, final follow-up; cSVA, C2–7 sagittal vertical axis.

* Significance between the two groups, *P* < .05.

** Significance between the two groups, *P* < .01.

*** Significance compared with preoperative, *P* < .05.

**Table 4:** Comparison of C7S and T1S between the two groups.

	Pre	Post	OM	FFU
C7S (°)				
Group A	23.36 ± 8.78	27.47 ± 7.13	27.34 ± 6.60	26.61 ± 8.00
Group B	25.13 ± 6.42	25.38 ± 6.98	24.37 ± 7.49	23.52 ± 7.47
*P*-value	0.319	0.213	.083	.094
T1S (°)				
Group A	26.07 ± 7.60	30.79 ± 6.92*	30.69 ± 7.99*	29.67 ± 8.07
Group B	27.85 ± 6.64	28.76 ± 7.13	28.12 ± 8.24	26.72 ± 7.37
*P*-value	0.289	0.228	0.187	0.108

Pre, preoperative; Post, postoperative; OM, one month after surgery; FFU, final follow-up; C7S, C7 slope; T1S, T1 slope.

* Significance compared with preoperative, *P* < .05.

## Discussion

Surgical methods for multilevel cervical diseases include multilevel ACDF and open-door LP. Surgeons need to take many aspects into account to choose the appropriate approach, such as the etiology of the disease, degree of the deformity, neurological impairment, comorbidities and patient’s expectations. Many studies focused on the comparison of these two approaches. Multilevel ACDF achieves direct spinal canal decompression, cervical spine stability, and restoration of the cervical spine curvature. The open-door LP is more suitable for patients who cannot be treated with the anterior approach with diseases such as ossification of the posterior longitudinal ligament. It enables direct posterior decompression and leads to indirect anterior decompression due to spinal cord palsy. The aim of both surgical methods is to achieve complete decompression of the spinal canal, and both have been developed as mature surgical procedures for the treatment of diseases including cervical spondylosis and spine cord injury. However, CSB is a special topic in a few studies in the literature. In recent years, scholars have noticed the value of CSB for cervical surgery. Studies have emphasized the importance of CSB before and after surgery, which may be closely related to clinical prognosis. However, the literature on the effects of improving or preserving CSB remains controversial. Many details of CSB and the two approaches need to be further clarified.

The efficacy of anterior and posterior approaches in improving CSB is still a matter of discussion. A mini-review is presented in Table 5 (11–17). The assessment of postoperative CSB includes two aspects: cervical spine curvature reconstruction and lordosis maintenance. ACDF restores the curvature of the cervical spine, and multilevel ACDF may make cervical spine more lordotic (14). A review of prospective cohort studies by Sakai et al. found that cervical alignment and balance improved after ACDF but worsened after LP (13). Some studies reported that the multilevel anterior approach has advantages over the posterior approach mainly in terms of spine reconstruction (14). ACDF restores cervical alignment by pulling the vertebrae forward (18). Previous studies showed that ACDF was able to significantly improve overall and segmental lordosis by changing the parameters of CSB (12). In addition, ACDF may improve cervical muscle function by altering cSVA so as to reduce the incidence of postoperative axial symptoms. However, ACDF may be inferior to LP in maintaining lordosis. It was reported that some of the parameters of CSB after multilevel ACDF were lower than those after single-level ACDF (12, 14). It appeared that as the number of levels increased, the capacity to maintain lordosis decreased (14). Unlike ACDF, LP causes damage to the posterior cervical column and the muscle-ligament complex, leading to partial loss of the weight-bearing function and disorder of sagittal balance (16, 19). Cervical spine has a tendency to tilt forward after LP, resulting in the need for greater muscle strength at the back of the neck to maintain an upright neck. Therefore, continuous muscle contraction is required to maintain proper neck position, and further, muscle spasm may occur, which is an important cause of postoperative axial pain (16). It was also reported that LP reduces lordosis and straightens the cervical spine (19). Although CSB appears to be less important than lumbar-pelvic balance, it is worth noting that changes in lumbar-pelvic balance probably result from adjustments in body posture to accommodate cervical sagittal imbalance (20–23).

**Table 5:** Mini-review on the anterior/posterior approach and CSB.

First author	Year	Approach	Follow-up results and period
Aita (11)	2000	LP	LP diminished lordosis and straightened the cervical spine. The range of motion and lordosis continued to decrease at a diminishing rate (mean 6.7 years)
Gillis (12)	2016	ACDF	Improvement in cervical lordosis was created and maintained. Increasing the number of levels resulted in improved cervical sagittal parameters (1 year)
Sakai (13)	2016	ADF/LP	CSB was maintained after ADF but deteriorated after LP (1 year)
Liang (14)	2019	ACDF/LP	ACDF showed a better balance-correcting ability but a poorer lordosis-preserving ability than LP (18 months)
Zhou (15)	2020	ACDF/ACCF	ACDF achieved more improvement in lordosis than ACCF with higher T1S (more than 12 months)
Pan (16)	2020	LP	Postoperative SVA increased after surgery, and the neurological function also improved (24 months)
Zhang (17)	2020	LP	The loss of cervical curvature after surgery was prone to occur when C7S was more than 20° (mean 24.9 ± 10.3 months)

ADF, anterior decompression with fusion; ACDF, anterior cervical discectomy and fusion; ACCF, anterior cervical corpectomy and fusion; LP, laminoplasty.

Spine sagittal balance plays an important role in the alignment against the axis of gravity with a minimum of energy expenditure (24). Spine sagittal imbalance is a significant factor not only for diseases but also for the planning of the perioperative period. CSB explains the posture of the cervical spine in the plane of sagittal, which shows the association between spine structure and relevant diseases. Cervical sagittal parameters are measured to evaluate cervical spine disorders and reflect the effect of surgery. Cervical lordosis is one of the major aspects of CSB. Parameters such as CCD, C2–7 angle, cSVA, C7S, and T1S were included in this study.

Cervical curvature is an important indicator of CSB. Borden et al. published the first study on cervical alignment in 1960 and proposed CCD to reflect cervical curvature (25). The advantage of this method is to minimize the impact of vertebral degeneration, but differences in bone and x-ray magnification are factors that affect the accuracy of measurement. For asymptomatic patients, restoring the curvature of the cervical spine through conservative treatment has a positive effect on preventing further degeneration of adjacent segments and even the entire cervical spine. For symptomatic patients, reconstructing the curvature of the cervical spine through cervical spine surgery can indirectly decompress the spinal canal and better restore the volume of the spinal canal. The Sagittal Cobb angle is clinically used in the measurement of spinal curvature, preoperative preparation, and postoperative evaluation for spine diseases. The C1–7 angle and C2–7 angle are measured for cervical lordosis because of their intra and interrater reliability and feasibility (26). Compared with the C2–7 angle, the C1–7 angle overestimates the lordosis of the cervical spine due to the hyperextension of C1 and C2 vertebrae. Therefore, the C2–7 angle is widely used in research studies on the cervical spine. cSVA is a classic sagittal parameter for assessing cervical spine alignment, which is closely related to cervical diseases and health-related quality of life. Studies have found that energy consumption increases as the center of gravity shifts, which indicates the importance of cSVA especially after cervical spine reconstruction. It was also reported that increased cSVA was associated with elevated intramedullary pressure in the spinal cord and was positively correlated with NDI scores (27, 28). In addition, regression studies show that T1S has a correlation with cSVA (15). The C7S and T1S are useful measurements for assessing CSB due to their great utility when full-length films are not available. Scholars tend to use T1S in their studies because C7S is strongly correlated with T1S, and the change of T1S in the pathological state is more obvious (29). Previous studies have shown that patients with T1S under 25° may have better CSB. However, patients with T1S over 25° are not meant to suffer from congenital or degenerative cervical diseases (30).

In our study, we compared 3-level ACDF with open-door LP from many perspectives, especially CSB. In terms of perioperative parameters, blood loss and drainage volume at 1 day after surgery in Group A was significantly less than that in Group B, indicating that 3-level ACDF had the advantages of small incisions, less trauma, and quick recovery. Therefore, the hospital stay of Group A patients was undoubtedly shorter than that of Group B patients. In addition, a significant difference was found in the NDI and JOA scores between the two groups postoperatively and at 1 month after surgery. Patients after 3-level ACDF recovered faster than open-door LP mainly in terms of function and life quality. However, there was no significant difference between the two groups with regard to NDI and JOA scores at FFU, which suggests that 3-level ACDF may benefit patients in the early stage of rehabilitation and there may be no difference between the long-term prognosis of the two surgical approaches. In terms of radiographic outcomes, patients after 3-level ACDF in our study reconstructed their cervical spine curvature and improved their cervical lordosis compared with those after open-door LP. The CCD of Group A increased significantly after surgery, indicating that 3-level ACDF increased cervical lordosis, while open-door LP did not change CCD significantly. In addition, 3-level ACDF showed advantages over open-door LP in terms of improving cervical spine curvature. Similar to CCD, the C2–7 angle increased significantly after 3-level ACDF, showing that cervical lordosis increased compared with open-door LP. No significant difference was observed in cSVA between both groups. From the results, conclusions can be drawn that the center of gravity did not shift significantly after 3-level ACDF and open-door LP, which guaranteed normal energy consumption in maintainin posture. Furthermore, T1S increased significantly after 3-level ACDF, which, in turn, increased cervical lordosis. Some studies have put forward the hypothesis that an increase of T1S may be a risk factor that aggravates symptoms, and therefore, reducing T1S may be beneficial to clinical recovery (30, 31). Also, it has been reported that excessive T1S may lead to spinal cord compression and cervical spondylosis (32). However, controversy still remains. T1S increases with age, and increased T1S may not be an exact variable to demonstrate clinical outcomes. Some scholars have attempted to use ratios such as C7S/T1S and Neck Tilt/T1S to replace T1S in their studies (29). More studies are needed to reveal the relationship between T1S and clinical prognosis.

This study had its limitations. Firstly, it was a retrospective comparative study. Secondly, the sample size was relatively insufficient. Thirdly, the research did not study further factors that affected cervical sagittal parameters. Therefore, a prospective randomized controlled study and a long-term follow-up with a large sample size are badly needed to analyze different cervical sagittal parameters after multilevel cervical spine surgery.

## Conclusions

Patient-reported clinical outcomes improved after 3-level ACDF and open-door LP compared with those before surgery according to NDI and JOA scores. However, clinical outcomes showed a significant difference between the two groups postoperatively and at 1 month after surgery, indicating that patients who underwent 3-level ACDF benefited more in the early stage of rehabilitation. From a long-term perspective, patients’ clinical outcomes showed no significant difference. From radiographic outcomes, 3-level ACDF had the advantages of reconstructing cervical lordosis over open-door LP with increased CCD and the C2–7 angle. However, cSVA, C7S, and T1S were analyzed with no significant differences. Given the limited literature on the comparison of multilevel cervical spine surgery, more research studies are recommended to elucidate the relationship between CSB and clinical and radiological outcomes.

## Data Availability

The data that support the findings of this study are available from the corresponding author upon reasonable request.
